# Indirect hemagglutination assay for diagnosing brucellosis: Past, present, and future

**DOI:** 10.14202/vetworld.2024.811-819

**Published:** 2024-04-15

**Authors:** M. M. Mikailov, Sh. A. Gunashev, E. A. Yanikova, A. A. Halikov, Aitbay K. Bulashev

**Affiliations:** 1 Laboratory of Infectious Pathology of Farm Animals, Caspian Zonal Research Veterinary Institute, Makhachkala, Dagestan, Russia; 2 Department of Microbiology and Biotechnology, Faculty of Veterinary and Livestock Technology, S. Seifullin Kazakh Agrotechnical Research University, Astana, Kazakhstan

**Keywords:** brucellosis, diagnostic value, indirect hemagglutination assay, prospects for improving the test, serological tests

## Abstract

Brucellosis is a zoonotic disease that causes enormous losses in livestock production worldwide and has a significant public health impact. None of the brucellosis-free countries is currently able to guarantee their ability to prevent the introduction of the pathogen due to the increase in tourism and the expansion of migration. The timely identification of infected animals is an effective means of preventing brucellosis and minimizing the epidemiological risk. The tube agglutination test, Rose Bengal plate test, complement fixation test, and enzyme-linked immunosorbent assay, which are routinely used to identify seropositive productive animals, have limitations and results that do not always correlate. The indirect hemagglutination assay (IHA) stands out among non-traditional methods because it is affordable, has a simple protocol, and is more reliable than classical serological tests, especially in cases of questionable and/or false-negative results. The diagnostic value of the IHA has long been studied by laboratories in several countries, but mostly by post-soviet research teams; therefore, the results continue to be published in Russian-language journals, ensuring that the local scientific community can access the results. In addition, the efficacy of this test for the diagnosis of brucellosis and other infectious diseases has not yet been reviewed. The purpose of this review was to summarize the results of studies on the development and use of IHA for the diagnosis of brucellosis and to determine the prospects for further improvement.

## Introduction

Brucellosis, the most common bacterial zoonosis, causes substantial livestock losses and poses a serious human health risk to both the food chain and professionals [1]. Central and South America, Africa, and part of Asia [2], as well as parts of the North Caucasus, Transcaucasia, Siberia, and Volga [3], are the most affected areas. Extensive world travel and the expansion of forced migration may lead to contact with the pathogen, even in brucellosis-free countries [4]. One of the main reasons for the lack of success in the fight against brucellosis is the low efficiency of serological tests used to identify infected animals. Three tests are most widely used for the serological diagnosis of brucellosis: the tube agglutination test (TAT), the Rose Bengal plate test (RBPT), and the complement fixation test (CFT). Enzyme-linked immunosorbent assay (ELISA) kits are currently available in the veterinary drug market [5]. Each of these texts has flaws, and their results do not always correlate. For example, TAT and its different variations do not detect non-agglutinating or incomplete antibodies, making them appear less sensitive and specific than any other standard test for all animal species. RBPT and its variants are performed in an acidic environment, which allows for the detection of both non-agglutinating and blocking (incomplete) antibodies; however, the prozone phenomenon causes strongly positive serum to appear negative in this test. CFT offers high sensitivity and specificity; however, it is a complex method that requires trained personnel. The applicability of this test is limited by its anti-complementary activity, the ability to detect only immunoglobulin (Ig)G1 antibodies, and the need for frequent reagent standardization [6–8]. Indirect ELISA protocols differ depending on the antigen, immunoconjugate (usually adapted to detect IgG1), and enzyme substrate. The same disadvantage applies to competitive ELISA tests in which a monoclonal antibody (mAb) competes with antibodies with low activity levels. In addition, these tests are difficult to perform because they require adequate budgets and well-equipped laboratories [9, 10]. Although ELISA is more sensitive than RBPT, brucellosis cannot always be detected in RBPT-positive animals [5]. Accurate, simple, and inexpensive tests that can be used in low-equity laboratories are required to eradicate brucellosis in developing countries [11].

The indirect hemagglutination assay (IHA) is a non-traditional serological test currently used for the diagnosis of brucellosis and has the greatest potential to be used in veterinary practice worldwide. It is a test that accurately detects brucellosis in animals at an early stage when conventional tests do not produce satisfactory or negative results [12, 13]. The test uses antigenic erythrocyte diagnosticum (Ag-ED), a red blood cell (RBC) with a surface-adsorbed antigen, to detect antibodies against *Brucella* in both blood serum and milk. Ag-ED reacts with specific blood or milk antibodies, causing RBCs to clump together and settle to the bottom of the tube as scalloped sediment. RBC sedimentation in the shape of buttons indicates a negative reaction. Antibody-coated erythrocyte diagnosticums (Ab-ED) are useful for detecting antigens in reverse. The diagnostic value of the IHA has long been studied by research teams in several countries but mainly by scientists of the former Soviet Union. As a result, the majority of the results are published in Russian-language journals. An exception is an article by Versilova *et al*. [14], which was published 50 years ago. Moreover, no peer-reviewed article on the efficacy of IHA in brucellosis and other infectious diseases is available.

The aim of this review was to summarize research findings on the development and use of IHA in the diagnosis of brucellosis and to determine its prospects for further improvement.

## Blood Serum-based IHA for Brucellosis Diagnosis

Since the 1970s, the diagnostic value of IHA for diagnosing brucellosis has been investigated. Several *Brucella-*sensitizing antigens, RBCs from various animal species, and methods for preparing Ag-ED and Ab-ED have been evaluated [15].

Chernysheva *et al*. [12] studied the specificity and sensitivity of IHA for the diagnosis of reindeer brucellosis based on sheep RBC (SRBC) sensitized to *Brucella* polysaccharides (PS) versus TAT. Blood serum samples were collected from healthy, experimentally infected, and spontaneously infected animals (n = 172). In the first test, infected animals were detected earlier than in the second test. Moreover, IHA antibody titers were 1.5–2 times higher in the challenged animals than in the TAT group. Hemagglutinins are detected more often than agglutinins in spontaneous reindeer infection.

Corbel and Day [16] used lipopolysaccharides (LPS) and intracellular proteins from *Brucella* *abortus* as Ag-ED. IHA was more sensitive and produced higher titers than standard tests (TAT, RBPT, and CFT) in the blood serum of known infected cattle. Significant titers were also observed in animals with no history of exposure to pathogens. A higher percentage of false positives and uncertain results was observed in cattle vaccinated with *B. abortus* 19 and 45/20. As a result of the absorption of heterologous antibodies from the test serum, there was a high correlation between the serological tests used.

Belchenko and Ivanov [17] compared the delayed CFT (DCFT) and TAT with IHA for the serological testing of unvaccinated calves raised on brucellosis-free and/or brucellosis-affected farms (n = 1158). Both tests were negative for brucellosis in calves from brucellosis-free farms. Brucellosis was detected in 3%, 2%, and 19% of the calves on farms with an acute infection outbreak using DCFT, TAT, and IHA, respectively. At the same time, the average IHA titer was twice as high as that of TAT. IHA detected normal agglutinins against SRBCs in calves’ blood serum in dilutions of 1:10–1:40. Preliminary absorption of test serum in a 50% suspension of non-sensitized SRBC allowed complete elimination of non-specific reactions. Hemagglutination at a dilution of ≥1:50 indicated a positive IHA result.

Versilova *et al*. [14] compared the diagnostic value of IHA based on *Brucella melitensis* and *B. abortus* LPS-sensitized SRBC with that of TAT using blood serum samples from 3519 people. Patients with chronic brucellosis (n = 524), those vaccinated with *B. abortus* l9 (n = 249), and residents from various infection foci (n = 2646) were included in the study. Agglutinins and hemagglutinin were detected in 30% and 43% of patients with chronic brucellosis, respectively. In some cases, the IHA titer reached 1:12,800, whereas TAT antibodies were detected primarily in the initial serum dilutions (1:100–1:200). IHA detected antibodies in vaccinated individuals with good sensitivity, and hemagglutinins were detected 2–3 times more frequently than agglutinins in the first 3 months following inoculation. Serological surveys of the population in three separate brucellosis foci showed that IHA had a higher number of reactors than TAT; hemagglutinin and agglutinin were identified in 40% and 22%, 12% and 22%, 11% and 5%, respectively. The sensitivity of IHA was also studied by testing the serum samples of cattle from brucellosis-free (n = 190) and brucellosis-affected farms (n = 246). Cattle were vaccinated with *B. abortus* 19 at 6–8 months of gestation. In animals from a brucellosis-free farm, the post-vaccination (p.v.) response rapidly decreased; thus, after 9–12 months, IHA was negative, and agglutinins and complement-fixing antibodies were found only in a few individuals. On the other hand, seropositivity persisted for a longer period of time on brucellosis-infected farm. The long-term persistence of hemagglutinin in vaccinated calves raised on brucellosis farms suggests that they were infected through contact with infection carriers.

Sadykov [18] described the efficacy of SRBC sensitization using *Brucella* extract produced by autoclaving bacterial cells. The following animal groups were used in this study: (i) cattle (n = 105) and deer (n = 44) kept in a fresh infection outbreak; (ii) experimentally infected cattle (n = 52) and sheep (n = 22); (iii) vaccinated and experimentally infected cattle (n = 142); (iv) vaccinated and revaccinated cattle (n = 577); and (v) healthy animals (n = 200). *Brucella* extracts were more effective than LPS or ultrasound antigens (SA) in identifying brucellosis-infected animals in all groups of animals with distinct epizootic characteristics. The antigen extract’s specificity was studied using the serum of animals (n = 137) with different diseases (ram epididymitis, tuberculosis, campylobacteriosis, pasteurellosis, toxoplasmosis, leukemia, viral abortion, tularemia, and swine fever). Negative results were observed in all cases. When blood serum samples from cattle (n = 2526), sheep (n = 74), and deer (n = 206) with varying brucellosis statuses were studied, positive results were achieved in a significantly larger proportion of animals using IHA (69%) than using plate agglutination (53%), TAT (33%), and CFT (31%). According to the authors, this test is particularly beneficial for new infection foci and brucellosis-free herds. Abusueva *et al*. [19] diagnosed acute, subacute, and chronic brucellosis in humans using an autoclaved bacterial suspension of *Brucella* vaccine strains treated with secondary sodium alkyl sulfate (SAS).

Taran *et al*. [20] developed methods for preparing *Brucella* Ag-ED, which consisted of SRBC loaded with the LPS-protein complex of *B. abortus* 19 cells [21]. The practical value of this test was determined for large populations of cattle (n = 6478) and sheep (n = 5540). All cattle in the brucellosis-free herds tested negative for TAT, CFT, and IHA but 0.8% of the sheep tested positive for pathogen-specific hemagglutinin. IHA detected a higher percentage of seropositive cattle at 3–4 months after vaccination with *B. abortus* 82 compared with standard tests. According to the authors, vaccinated animals lose agglutinating and complement-fixing antibodies faster than hemagglutinins. It was recommended that the analyte should be absorbed by normal SRBC to avoid false-positive IHA results caused by the presence of natural agglutinins in the test sera.

Renoux and Renoux [22] detected hemagglutinins in 99.8% of brucellosis-infected heifers (n = 968), but specific agglutinins and complement-fixing antibodies were detected in only 59% and 91%, respectively. In the analysis of human blood serum with positive TAT or CFT results (n = 778), hemagglutinins were found in only 20% of the samples; however, 3% of the patients were IHA negative. IHA revealed remote or undiagnosed human *B. abortus* infections in 29% of 18,367 randomly tested sera in rural areas. According to the authors, this “points to a high prevalence of undetected brucellosis in humans, especially in areas where cattle are raised”. A simple and easy-to-perform test could replace traditional tests for diagnosing brucellosis and conducting epidemiological survey. The high specificity of this test based on SRBC coupled with ABS isolated from *B. abortus* was demonstrated by the absence of a false-positive result in the study of 2000 healthy people and animals using blood serum. Unlike TAT and CFT, IHA did not produce a false-negative result in serological tests of 1800 serum samples from people and animals with confirmed brucellosis infection [23].

The diagnostic value of heat-extracted antigen-based IHA and CFT in rams (n = 10) infected with *Brucella* *ovis* through preputial inoculation was investigated. Observations were performed weekly for 1 year to monitor changes in clinical, bacteriological, and serological parameters. *B. ovis* was isolated from the semen of all rams 5 weeks post infection (p.i.). All animals developed significant CFT titers between 2 and 9 weeks p.i. Thereafter, CFT was a reliable indicator of infection in six, highly indicative in three, and unreliable in one ram, whereas all animals developed significant IHA titers at 8–10 weeks p.i., which persisted until the end of the experiment. IHA using heat-extracted antigens could serve as a routine diagnostic test for *B. ovis* infection in sheep [24].

The main disadvantage of IHA is the use of fresh RBCs as solid phases, which are fragile, susceptible to microbial contamination and hemolysis, and can only be stored for a short period of time. This problem can be overcome by stabilizing RBCs with certain chemicals so that the cells can be used for several months without significant loss of antigen-binding activity. Rai *et al*. [25] assessed the serodiagnosis of bovine brucellosis using formaldehyde, glutaraldehyde, pyruvic aldehyde, and double-aldehyde-stabilized SRBC coated with *B. abortus* 99 SA in IHA. Serum samples were collected from 23 TAT-positive cows, 12 clinically suspected but TAT-negative cows, and 20 healthy unvaccinated animals. Ag-ED stabilized with double-aldehyde and glutaraldehyde increased the test specificity over preparations stabilized with formaldehyde or pyruvic aldehyde, allowing IHA to clearly distinguish healthy cows from TAT-positive and clinically suspect animals. Moreover, SRBC treated with double-aldehyde made cells more accessible to antigen coating without activation of tannic acid compared with glutaraldehyde.

The sensitivity of IHA, based on cell-wall antigens extracted from *B. abortus* 19 by autoclaving and subsequent treatment with SAS, was studied in 18-month-old heifers (n = 16) that had been experimentally infected with virulent *B. abortus* 54 at a dose of 1×10^6^ cells. On the 10^th^ day p.i., all blood sera yielded negative results according to traditional tests; however, anti-*Brucella* hemagglutinins were detected in nine and five animals at titers of 1:50 and 1:100, respectively. All infected heifers had IHA antibodies in their blood serum, on the 20^th^ day p.i. (≥1:100). At this time, 11 and 3 heifers tested positive using TAT and CFT/DCFT, respectively. At the end of the trial (days 37–48), all animals tested positive using IHA (1:200–1:3200); however, in some heifers, antibodies were not detected using CFT (n = 3), DCFT (n = 2), or TAT (n = 1). Virulent strain cultures were isolated from all infected animals. Therefore, compared to conventional testing, IHA allowed more infected animals to be detected in the first 3 weeks p.i. [26].

The simplicity of RBC sensitization, reproducibility, and protocol simplicity, as well as the sufficient sensitivity of IHA, provided the foundation for the industrial production of IHA in the Russian Federation (RF). Since 2007, the “IHA-kit (Vetmedservice LLP, Republic of Dagestan, RF) for the diagnosis of cattle and sheep brucellosis” (“IHA-kit”) developed by the Caspian Zonal Research Veterinary Institute (Makhachkala, RF), Russian State Centre for Animal Feed and Drug standardization (Moscow, RF), and the All-Russian Research Institute for Animals Brucellosis and Tuberculosis (Omsk, RF) has been officially accepted by the Russian veterinary practice. GOST 34105-2017: “Animals. The current Interstate Standard is Laboratory diagnoses of brucellosis serological methods (Kazakhstan, Kyrgyzstan, Armenia, Russia, and Belarus)”. This standard sets out the requirements and rules for using IHA.

A significant number of cattle and sheep in the Rostov region of the RF were tested using the “IHA-kit.” Serological studies were performed on the blood serum of cattle kept in farms, which were divided into four categories: (i) brucellosis-free farms without vaccination (n = 15,790); (ii) brucellosis-free farms using revaccination of animals with *B. abortus* 82 (n = 4526); (iii) brucellosis-affected farms without vaccination (n = 13,878); and (iv) brucellosis-affected farms using revaccination of animals with *B. abortus* 82 (n = 2149). In addition, the serum from sheep in a brucellosis-free flock without vaccination (n = 20,167) was serologically examined. All cattle and sheep kept in brucellosis-free herds were negatively tested using TAT, CFT, and IHA-kit. In 12 herds from the second category, no IHAs were positive for animals with a titer of ≥1:400. In the remaining six herds, positive IHA (≥1:400) results were within 2%, and most animals showed positive TAT and CFT results at elevated titers of ≥1:200 and ≥1:20, respectively. These herds were considered to be infected with brucellosis. In farms where *B. abortus* 82 vaccine was not used (3^rd^ category), infection was detected in 6% and 7% of animals using TAT + CFT and the “IHA-kit” (≥1:100), respectively. In 96% of the cases, the combined positive results of TAT + CFT coincided with that of IHA. In one of the herds, eight aborted cows were positive for IHA, whereas no agglutinin or complement-fixing antibodies were detected in four. During planned diagnostic examinations on farms in the fourth category, most animals testing positive with TAT- and CFT exhibited IHA titers ≥1:200–1:400 [27].

The diagnostic value of the “IHA-kit” in comparison to TAT, CFT, and radial immunodiffusion with O-PS antigen (RID/O-PS) was investigated in 2256 sheep from 15 flocks across Siberia. The “IHA-kit” was a more specific and sensitive test for sheep brucellosis than traditional tests. It was recommended for screening sheep one year after a single subcutaneous vaccination with *B. abortus* 19 at a dose of 40 × 10^9^ cells or 4 months after primary conjunctival inoculation at a dose of 4 × 10^9^ cells [28]. In addition, the “IHA-kit” showed higher sensitivity than TAT, CFT, RBPT, and RID/O-PS, enabling the identification of an additional 5%–15% of brucellosis-infected animals in affected herds and offering a reliable diagnosis at the beginning of infection. The kit enabled the detection of the maximum number of infected cattle in an acute infection focus and, according to the authors, could replace the TAT + CFT (Shchelkovsky Biocombinat, Moscow, RF) combination [29]. We performed a comparative serological analysis of ewes from brucellosis-free (n = 496) and affected flocks (n = 559) where an acute infection outbreak was reported in the previous year using the “IHA-kit,” CFT, TAT, and RID/O-PS. In the first group, no responders were detected, whereas in the second group, anti-*Brucella* antibodies were detected in 28%, 20%, 13%, and 5% of ewes [30, 31]. In another flock (n = 423) where a brucellosis outbreak occurred, TAT, CFT, and RBPT missed 15%, 7%, and 8% of IHA-seropositive ewes, respectively [32]. The following section compares the specificity and sensitivity of the kit to the commercial ELISA kits Sibbiotest (Novosibirsk, RF) and Biok (Kursk Biofactory, RF). Fifty-two non-immune cows from brucellosis-free farm and 82 vaccinated cows from a brucellosis-infected farm were tested. The diagnostic kits showed negative results when tested on animals from farms without infection. When testing cows from a brucellosis-infected farm, the “IHA-kit” was less sensitive than the Sibbiotest but more sensitive than the Biok-kit [33]. However, IHA was more sensitive than ELISA tests and RID/O-PS during sheep serological study [34]. In our previous study, the “IHA-kit” showed comparable results to the TAT and International Diagnostic Exchange (IDEXX) Brucellosis Serum Ab Test (Westbrook, Maine, USA). The proportions of cattle with anti-*Brucella* hemagglutinins (13%) and agglutinins (11%) were similar to those of ELISA-positive animals (12%); however, complement-fixing antibodies were found in only 8% of the animals [35].

IHA is a simple and reliable test that has attracted the interest of researchers from other brucellosis-affected countries. Mohan *et al*. [36] (Veterinary and Animal Sciences University, Ludhiana, India) reported that agglutination-based tests had a higher diagnostic yield than ELISA. The average titers of cattle naturally infected with brucellosis (n = 15) and healthy analogs (n = 6) vaccinated as calves with *B. abortus* 19 differed significantly according to the IHA, TAT, and microagglutination test (MAT), whereas there were no significant differences between the groups using ELISA. In another study [37], they investigated the effects of bacteriophage therapy on the immune responses of naturally infected cows. IHA detected the highest antibody titer in infected cows on day 0, and toward the end of the experiment, its sensitivity was significantly higher than that of ELISA and MAT. Li and Su [38] (Animal Husbandry and Veterinary Station of Pingdingshan City, China) conducted serological surveys on white goats for brucellosis (n = 563), foot-and-mouth disease, and paratuberculosis using IHA in conjunction with TAT, the Tiger Red Antigen Plate Test, and ELISA. The results of the IHA were comparable to those of other agglutination phenomenon-based tests.

## Milk-based IHA for Brucellosis Diagnosis

Milk is an alternative biological fluid for serological testing of brucellosis in ruminants, and it can be easily collected without the use of special equipment [39]. Milk samples are preferable to blood serum as a non-invasive biological material for many infectious diseases in dairy animals, since they allow for the determination of the health status of not only individual animals but also the whole herd with minimal material costs. The milk ring test (MRT) is a recognized method in RF for the diagnosis of brucellosis in non-vaccinated cattle and is considered to be sensitive [40]. At the same time, MRT sometimes produces non-specific positive results, but much more frequently produces a negative reaction in cows infected with brucellosis, which is associated with factors such as milk fat content or acidity and mastitis [9]. MRT did not succeed in diagnosing brucellosis in sheep and goats compared with cows. It has been suggested that small fat globules in sheep and goat milk less actively absorb agglutinated stained *Brucella* cells (antigen for MRT), do not rise to form a typical colored ring, and precipitate at the bottom of the tube [41]. It has been demonstrated that using IHA for milk (IHA/m) has specificity, and its results are unaffected by clinical or subclinical mastitis or pregnancy status. Culture isolation and polymerase chain reaction (PCR) results were positive in 67% and 83% of cases in cows with milk hemagglutinin. The cattle of brucellosis-free farms in the early period (1–2 months) post vaccination (p.v.) with *B. abortus* 82 were negative using IHA/m, indicating that animals can be tested for brucellosis on the 30^th^-day p.v., as opposed to TAT and CFT, which are used no earlier than 6 months p.v. [42]. IHA/m had a higher sensitivity than MRT when testing the milk of cows from brucellosis-affected farms, and it was proposed as a reconnaissance test for monitoring the epizootic situation in unvaccinated and immunized brucellosis-free herds, as well as for identifying animals with localization of the pathogen in the mammary gland [43]. In view of the low proportion of healthy animals with IHA/m titers ≥1:50, this indicator should be considered a positive result. MRT and IHA/m results agreed in 84% of cases when studying the milk of cows from brucellosis-affected herds. Antibodies were detected using IHA/m and MRT in four and two samples, respectively, in five PCR-positive milk samples [44].

Using the “IHA-kit,” we simultaneously analyzed milk and blood serum samples of cows for anti-*Brucella* antibodies. MRT, TAT, and CFT were used to examine the same milk and serum samples. Analyses were performed in cows from brucellosis-free (n = 400) and affected (n = 880) farms. All paired samples from cows in the first group were negative, but MRT was positive in four mastitis animals. In the second group, IHA/m had comparable sensitivity to its blood serum counterpart (IHA/s) and outperformed MRT, TAT, and CFT. Milk hemagglutinins, for example, were detected in 28% of the cows, which also reacted positively with IHA/s, whereas pathogen-specific antibodies were detected in only 14% of the animals using MRT. Anti-*Brucella* agglutinins and complement-fixing antibodies were detected in 18% of the cattle. It was discovered that IHA/m has high sensitivity and is suitable for identifying infected animals at an early stage of the disease when testing cows that were aborted owing to brucellosis [45]. Subsequently, the diagnostic value of IHA/m was compared with a commercial ELISA (Biok, Kursk Biofactory) and MRT. This study included 37 paired milk and blood serum samples from healthy cows and 23 similar analytes from a farm with brucellosis but no subclinical mastitis. Pathogen-specific antibodies were not detected in the blood serum or milk of the first group of cattle. However, milk antibodies were detected in 26% and 61% of samples using MRT and IHA/m, respectively, and blood serum antibodies were detected in 44% and 74% of samples using ELISA and IHA/s, respectively [46].

In a study by Gulieva *et al*. [47], IHA/m was more informative than IHA/s, MRT, TAT, and CFT when testing four heifers 7 days after abortion. The IHA/m results were positive up to titers of 1:128–1:512; however, the MRT was only weakly positive in one heifer, whereas it was doubtful in the other. No agglutinins were detected in the blood serum, but two cows had hemagglutinins and complement-fixing antibodies. According to the authors, the relatively high sensitivity of IHA/m may be associated with the development of an infectious process in the mammary glands, resulting in IgA production by regional lymph nodes.

In our previous study [48] we compared the “IHA-kit” to MRT, TAT, and CFT using milk and serum from small ruminants (n = 443) from brucellosis-affected flocks. The kit gave positive results for both analytes in 7% of the animals, whereas anti-*Brucella* antibodies were detected in only 3% of sheep and goats. On the basis of these findings, we conclude that small ruminants infected with brucellosis can be identified in the early stages of brucellosis by detecting hemagglutinin in their milk.

## Use of IHA for *Brucella* Detection

Culture isolation remains the “gold standard” for diagnosing brucellosis. It can take up to a month, and it is frequently impossible to isolate the pathogen from individuals with positive serological results or clinical signs of the disease; therefore, negative results of culture isolation are of minor importance [49]. Therefore, *Brucella*-specific mAbs are considered to be promising tools for detecting pathogens [50, 51]. Previously, we obtained two types of mAbs specific to *Brucella* poly-B antigen conjugated to SRBC using amidol. Using these mAbs as SRBC sensors allowed for a more precise differentiation of *B. abortus* and *B. melitensis* from other closely related bacteria (*Yersinia*, *Salmonella*, and *Francisella* spp.) than using rabbit polyclonal antibodies [52]. We used pAb-ED-based IHA and PCR to detect *Brucella* in milk, lymph nodes, and organ samples from four aborted ewes. Pathogen antigens and DNA were detected in all tested animals, and the results of direct diagnostic methods were confirmed using IHA/m, IHA/s, and CFT [45]. pAb-ED was prepared using anti-*B. ovis* antibody and tested for diagnostic value using biomaterial samples (n = 52) from 10 rams in comparison with antibody neutralization test (ANT) and culture isolation [53]. These animals came from a farm with a long history of infectious epididymitis. Cultures were isolated from one-quarter of the samples examined; however, pathogen antigens were detected in 60% and 45% of cases using pAb-ED/IHA and ANT, respectively. No pathogen antigens were detected in biomaterials (n = 300) from healthy rams (n = 10), or ewes (n = 60) vaccinated with *B. melitensis* Rev-1. A sensitivity of 1 × 10^6^ – 3 × 10^6^ cells/mL was observed, similar to the findings of Koshkidko *et al*. [54], who used RBC sensitized with antibodies against *Brucella* spp. and *Francisella tularensis*. At present, IHA, together with modern molecular biology (PCR, Loop-mediated isothermal amplification) and immunology (ELISA, immunofluorescence assay) methods, is used to identify *Yersinia pestis* [55] and *Mycoplasma ovipneumoniae* [56].

## Conclusion

ED-based IHA tests are simple, quick, easy to perform, and reproducible and can be used to diagnose brucellosis and other potentially fatal infectious zoonotic diseases. The main disadvantage of IHA is the insufficient specificity of antigens used to coat RBC [15]. *Brucella* surface antigens, primarily LPS, have an antigenic structure similar to that of other closely related bacteria [57–59]. However, the specificity of Ag-ED prepared using various *Brucella* antigens has not been investigated. Zheludkov *et al*. [60] examined serum samples from 270 patients with chronic brucellosis using IHA and found that anti-*B. abortus* 99 hemagglutinins cross-reacted with *Yersinia enterocolitica* 09 hemagglutinin. False-positive IHA results have also been reported in patients who have been infected or vaccinated against tularemia as well as patients with other infectious diseases using RBCs sensitized with *B. abortus* 19 LPS-protein antigens, which has also been explained by the commonality of surface antigenic determinants of *Brucella* and related pathogens [20].

False-positive IHA results have also been reported in cases of bovine tuberculosis [61–63], sheep and goat mycoplasmosis [56], melioidosis [64], and cystic echinococcosis [65, 66]. Armstrong *et al*. [67] found significant differences in the results of three Australian centers using IHA to diagnose melioidosis, despite testing the same samples.

The standardization and widespread use of IHA in diagnostic practice are possible with the availability of a protein that is strictly specific for a particular pathogen, as well as technology that allows it to be generated in unlimited quantities with constant properties. At present, several studies have demonstrated the excellent specificity of the recombinant outer membrane and periplasmic proteins [68–70], the diagnostic potential of which has been described in our recent review [71]. These homogeneous and easily standardized proteins can be assessed as RBC sensitins to identify the most suitable antigen for IHA.

The use of IHA for the diagnosis of brucellosis in the p.v. period remains poorly studied, and the current data contradict each other, most likely due to differences in vaccine types and injection methods, animal species, and antigen nature [14, 20, 28, 42]. We cannot ignore the suggestion that individual *Brucella* proteins could be used as novel antigens to distinguish infected from vaccinated animals [72, 73], which is the primary issue in endemic regions. Therefore, identifying proteins that allow IHA to distinguish between post-infectious and post-vaccinal antibodies should be regarded as one of the most important problems in brucellosis serodiagnosis.

The development of simple, inexpensive, sensitive, and specific rapid methods for detecting pathogens in an analyte is an important task in veterinary medicine in brucellosis-prone developing countries. One promising approach to resolving this problem is the use of a homogeneous mAb against *Brucella* as an RBC-sensitizing Ig that can also be standardized and produced in unlimited quantities.

Removing agglutinins from the test serum of heterologous erythrocytes complicates the procedure and increases the time required for testing. It is preferable to use RBCs from the same species as the serum under examination to avoid these issues. IHA has uneven sensitivity and specificity when erythrocytes from animals of the same species are used [15]. Moreover, it is worth noting that research on the use of avian erythrocytes as an indicator, such as IHA using chicken RBC, has shown comparable sensitivity and specificity to human O-cell-based IHA in diagnosing amebiasis [74].

Thus, IHA has not yet been widely used in the diagnosis of brucellosis; however, given the availability of standardized *Brucella* recombinant protein(s) or pathogen-specific mAb(s), it has the potential to become a specific immunological test not only for serological diagnosis but also for pathogen identification. The standardization of Ag-ED or Ab-ED, as well as the timing of use of IHA in the p.v. period, will enable the differentiation of immune individuals from infected animals. This is critical in developing countries where brucellosis has become endemic and the low reliability of traditional diagnostic methods decreases the efficacy of anti-epizootic interventions aimed at eradicating infection.

## Authors’ Contributions

AKB and MMM: Conceptualized and designed the study, conducted a comprehensive literature search, analyzed the gathered data, and drafted the manuscript. SAG, EAY, and AAH: Conducted the final revision and proofreading of the manuscript. All authors have read, reviewed, and approved the final manuscript.
